# Outcome of Did Not Attend Outpatient Appointment

**DOI:** 10.1192/bjo.2022.479

**Published:** 2022-06-20

**Authors:** Kalsum Saleh, Annolusola Ige

**Affiliations:** Essex Partnership NHS Foundation Trust, Loughton, United Kingdom

## Abstract

**Aims:**

Failure to attend outpatient clinic appointments by service users without prior notification is a major contributor to waste resources. Failure to attend earlier in treatment predicts attrition later in treatment (Goode, 1997; Aubrey et al, 2003) leading to further waste of resources. The department of health figures for England show that failure to attend outpatient clinic is more in mental health clinics (19.1%) compared with overall figures for other specialties 11.7% (Department of Health, 2003). Lack of appropriate follow-up when a service user does not attend as appointment has been identified as a contributory factor in Serious Incident investigations, Domestic Homicide Reviews and Safeguarding Adults Reviews. Our aim of this study is to see if we are adherent to trust policy or not.

**Methods:**

A questionnaire tool was designed by using trust guidelines regarding DNA appointment

- Was The DNA Recorded in patient's records? YES/NO
- Was the information (DNA) shared with GP? YES/NO
- Was The DNA discussed in MDT meeting? YES/NO
- For new referrals was the referrer involved in review and decision of next step? YES/NO
- Were alternative venues considered for carrying out the assessment to support the person to engage, e.g. GP Surgery? YES/NO

Data were collected by team and analysed by Dr Saleh using electronic records.

**Results:**

88 outpatient appointments were flagged as DNA appointments between 1 April 2021 to 31st May 2021

1. In 87.5% cases DNA was recorded in patient's records?
2. In 45.45% cases the information (DNA) was shared with GP
3. In 45.45% cases DNA was discussed in MDT meeting
4. In 0% case the referrer was involved in review and decision of next step
5. In 11.36% cases alternative venues was considered for carrying out the assessment to support the person to engage, e.g. GP Surgery.
6. 25 patients DNA appointment twice

**Conclusion:**

We are not adherent to trust policy.

